# Supplementary material for: Identification of Cell-Surface Proteins Endocytosed by Human Brain Microvascular Endothelial Cells In Vitro
Source: Pharmaceutics. 2020 Jun 23;12(6):579. doi: 10.3390/pharmaceutics12060579 (PMC7356521; doi:10.3390/pharmaceutics12060579)
Supplement: Supplementary file 1 [file pharmaceutics-12-00579-s001.zip › pharmaceutics-805541 - supplementary/Supplementary Materials_Table S1_Figure S1-S3_200623_proof.docx]

Supplementary Materials: Identification of cell-surface proteins endocytosed by human brain microvascular endothelial cells in vitro

Shingo Ito, Mariko Oishi, Seiryo Ogata, Tatsuki Uemura, Pierre-Olivier Couraud, Takeshi Masuda and Sumio Ohtsuki

**Table S1.** Number of identified biotinylated cell-surface and internalized proteins in hCMEC/D3 cells and HUVECs.

| **Cell lines** | **Number of proteins** | | |
| --- | --- | --- | --- |
|  | **Total identified** | **Biotinylated**  **cell-surface** | **Biotinylated endocytic cell-surface** |
| hCMEC/D3 | 563 | 378 | 125 |
| HUVEC | 399 | 225 | 113 |


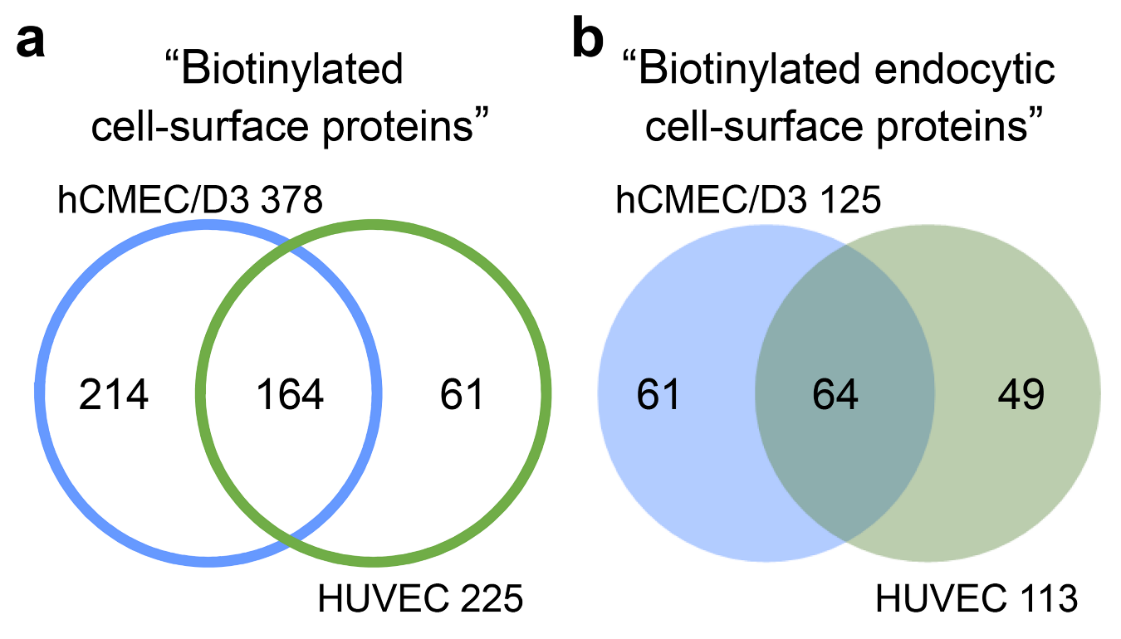


**Figure S1.** Comparison of the number of “biotinylated cell-surface proteins” **(a)** and “biotinylated endocytic cell-surface proteins” **(b)** between hCMEC/D3 cells and HUVECs. **(a)** “biotinylated endocytic cell-surface proteins” between hCMEC/D3 378 and HUVEC 225; **(b)** “biotinylated endocytic cell-surface proteins”between hCMEC/D3 125 and HUVEC 113.


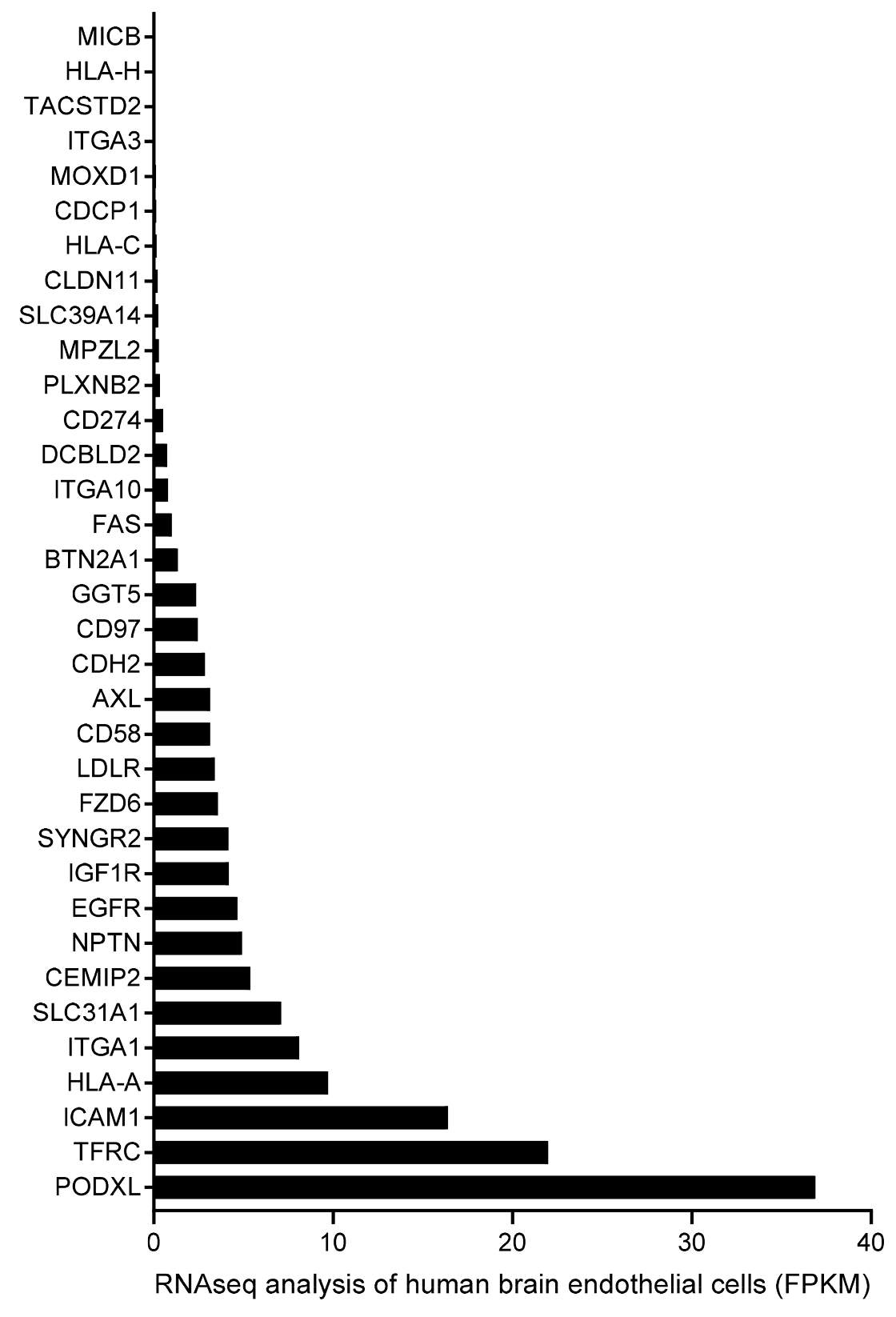


**Figure S2.** The mRNA expression levels of 34 proteins identified in human brain endothelial cells. The mRNA data are from the open database of mRNA expression levels in human endothelial cells [21].


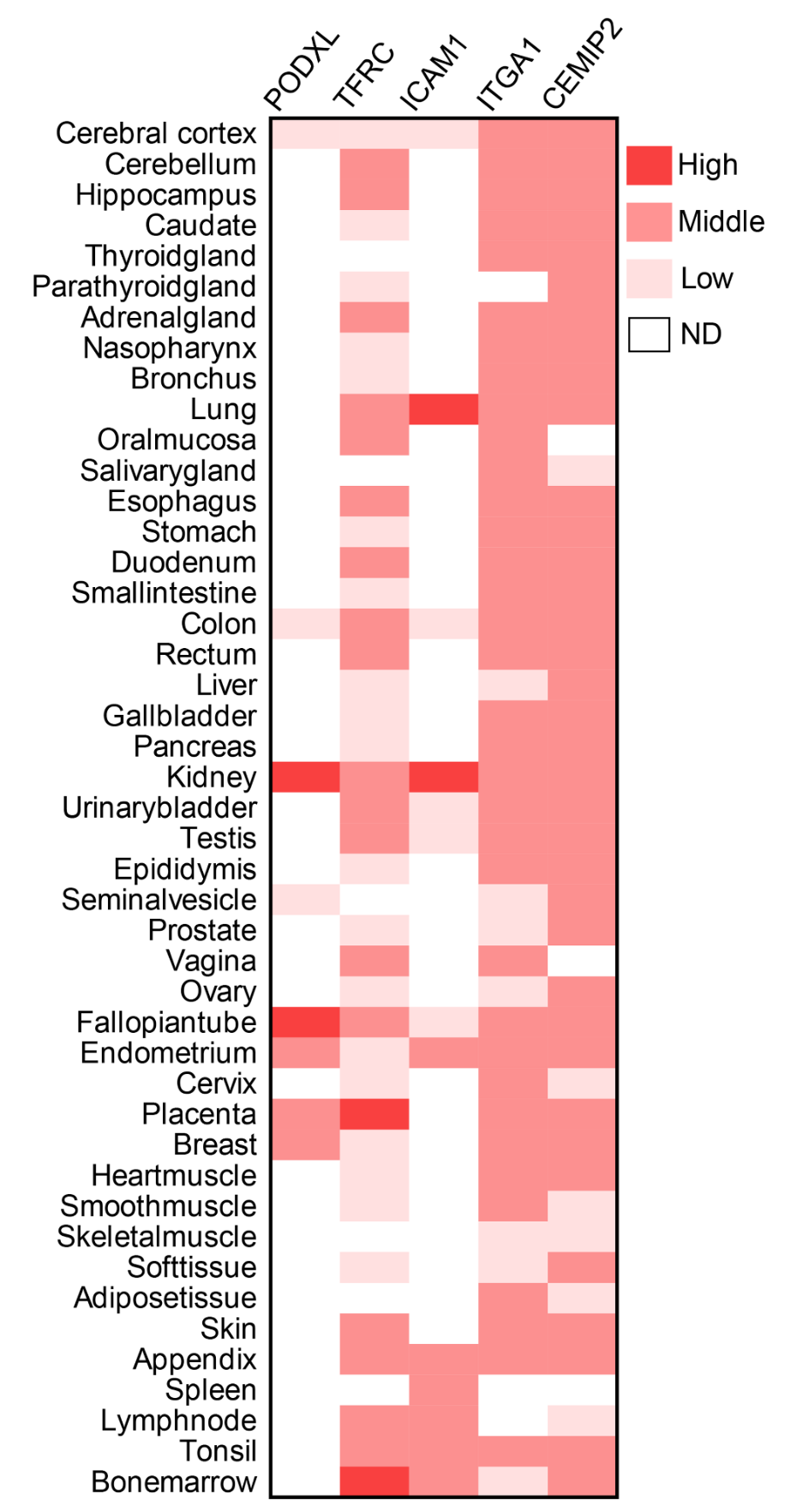


**Figure S3.** Localization, in human tissues, of the five proteins that we selected from those identified in this study using immunohistochemistry. These data are from the human protein atlas [19,20]. ND: not detected.
